# RGD-Peptide Functionalization Affects the *In Vivo* Diffusion of a Responsive Trimeric MRI Contrast Agent through Interactions with Integrins

**DOI:** 10.1021/acs.jmedchem.1c00264

**Published:** 2021-05-07

**Authors:** Giuseppe Gambino, Tanja Gambino, Liam Connah, Francesca La Cava, Henry Evrard, Goran Angelovski

**Affiliations:** † Max Planck Institute for Biological Cybernetics, Department for Physiology of Cognitive Processes, Max-Planck-Ring 11, 72072 Tübingen, Germany; ‡ Nathan S. Kline Institute for Psychiatric Research, 140 Old Orangeburg Road, Orangeburg, New York 10962, United States; § Werner Reichardt Centre for Integrative Neuroscience, University of Tübingen, Otfried-Müller-Strasse 25, 72076 Tübingen, Germany; ∥ Laboratory of Molecular and Cellular Neuroimaging, International Center for Primate Brain Research (ICPBR), Center for Excellence in Brain Science and Intelligence Technology (CEBSIT), Chinese Academy of Sciences (CAS), Shanghai 200031, PR China

## Abstract

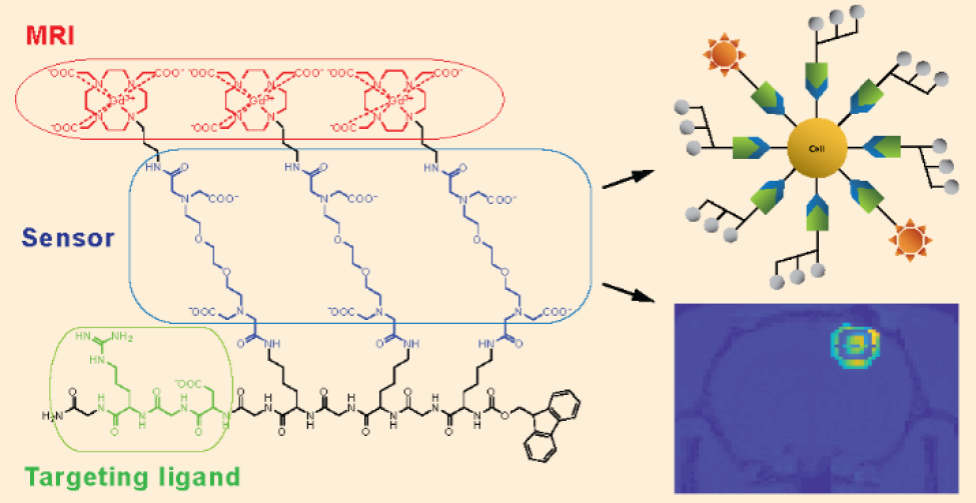

The relevance of MRI as a diagnostic methodology has been expanding significantly with the development of molecular imaging. Partially, the credit for this advancement is due to the increasing potential and performance of targeted MRI contrast agents, which are able to specifically bind distinct receptors or biomarkers. Consequently, these allow for the identification of tissues undergoing a disease, resulting in the over- or underexpression of the particular molecular targets. Here we report a multimeric molecular probe, which combines the established targeting properties of the Arg-Gly-Asp (RGD) peptide sequence toward the integrins with three calcium-responsive, Gd-based paramagnetic moieties. The bifunctional probe showed excellent ^1^H MRI contrast enhancement upon Ca^2+^ coordination and demonstrated a longer retention time in the tissue due to the presence of the RGD moiety. The obtained results testify to the potential of combining bioresponsive contrast agents with targeting vectors to develop novel functional molecular imaging methods.

## Introduction

The interest in molecular imaging methodologies and demand for their use increases significantly year after year due to their ability to help in the understanding of biological processes.^1^ Moreover, it is becoming clear that the ability to investigate physiological mechanisms in a noninvasive way could prompt an incredible leap forward in the understanding of the causes of many obscure diseases, such as neurodegeneration and cancer.^2^ Consequently, knowing the physical–chemical properties of the diseased tissues would enable their detection at much earlier stages or even prevent the full development of the disease itself.

Within the tools available for molecular imaging, MRI is certainly among the most promising.^3^ Due to its unlimited tissue penetration, lack of ionizing radiation and high spatial resolution, MRI is intrinsically capable of producing highly detailed images of soft tissues. Additionally, it is possible to dramatically increase the local signal with the use of CAs.^4,5^ These compounds, commonly organometallic complexes based on paramagnetic Gd^3+^ or Mn^2+^, are able to shorten the longitudinal and transverse relaxation times, *T*_1_ or *T*_2_, of the proton nuclei of the surrounding water molecules, remarkably altering the MRI signal in the tissue that they perfuse. The growing interest for molecular imaging prompted the development of a particular class of MRI CAs, referred to as “smart” or “responsive” CAs. Such probes can modulate their capability of enhancing the generated MRI signal in response to the physical–chemical properties of their environment.^6^ Several examples of such systems have been developed in the last years, showing an MRI signal response to enzymatic activity,^7,8^ pH,^9,10^ pO_2_,^11^ or endogenous metal ions^12^ such as Ca^2+^^13,14^ and Zn^2+^.^15^ Clearly, the possibility to monitor and possibly quantify these properties by using molecular probes holds excellent potential and can be used as the foundation of several noninvasive imaging methodologies. However, the translation of the use of these CAs into clinics would first require overcoming obstacles related to their *in vivo* application, such as their quantification, which is often precluded due to the fast diffusion of the probe.^16,17^

Different approaches have been adopted in attempts to address such problems, resulting in an exciting expansion of the responsive CAs field. One method involves the development of probes that target specific tissue components. By functionalizing conventional CAs with an appropriate moiety, it is possible to achieve selective binding, covalent or noncovalent, to a specific target.^18−20^ Such approaches can enable the imaging of a desired tissue by exploiting the overexpression of particular receptors and labeling the molecular probe with the appropriate vector.^21^ One widely used example for the application of this approach relies on the development of CAs functionalized with folic acid, which targets the overexpressed folate receptors in cancerous cells, thus resulting in a higher local concentration of the functionalized probe in the tumor tissue.^22,23^

To this end, peptide sequences have been increasingly used over the years in order to broaden the spectrum of biological markers available for targeting. Meanwhile, several sequences have been identified to specifically bind to a molecular receptor or interact with a particular protein or enzyme. Consequently, a number of systems have been reported where molecular probes of different sizes are functionalized with one or more targeting peptide units.^24,25^ Such systems were able to selectively accumulate or remain for longer periods of time in the tissue of interest.

Distribution and retention of responsive CAs in the targeted tissue is another major feature that has great room for improvement. Namely, some of the most interesting applications of molecular imaging would require recording the signal of the biomarker of choice over a certain period of time, possibly while maintaining a relatively constant concentration. The capability of the responsive probe to remain in the tissue long enough and thus keep the MRI signal constant due to its own slow diffusion represents a critical point for functional imaging studies. However, the vast majority of currently developed responsive CAs are usually smaller molecules (MW < 1.5 kDa), which diffuse and wash out of the tissue quite quickly, significantly limiting the time window for the imaging procedure. A common technique to overcome this issue is represented by the use of nanosized responsive CAs (MW > 10 kDa): these probes slowly diffuse in the tissue because of their size, although their biodegradability and long-term accumulation in the tissue may then become an issue. On the other hand, by functionalizing a smaller molecular probe with an appropriate peptide vector, it would be possible to obtain a more versatile system, without the issues related to the biodegradability of nanoparticles.

**Scheme 1:**
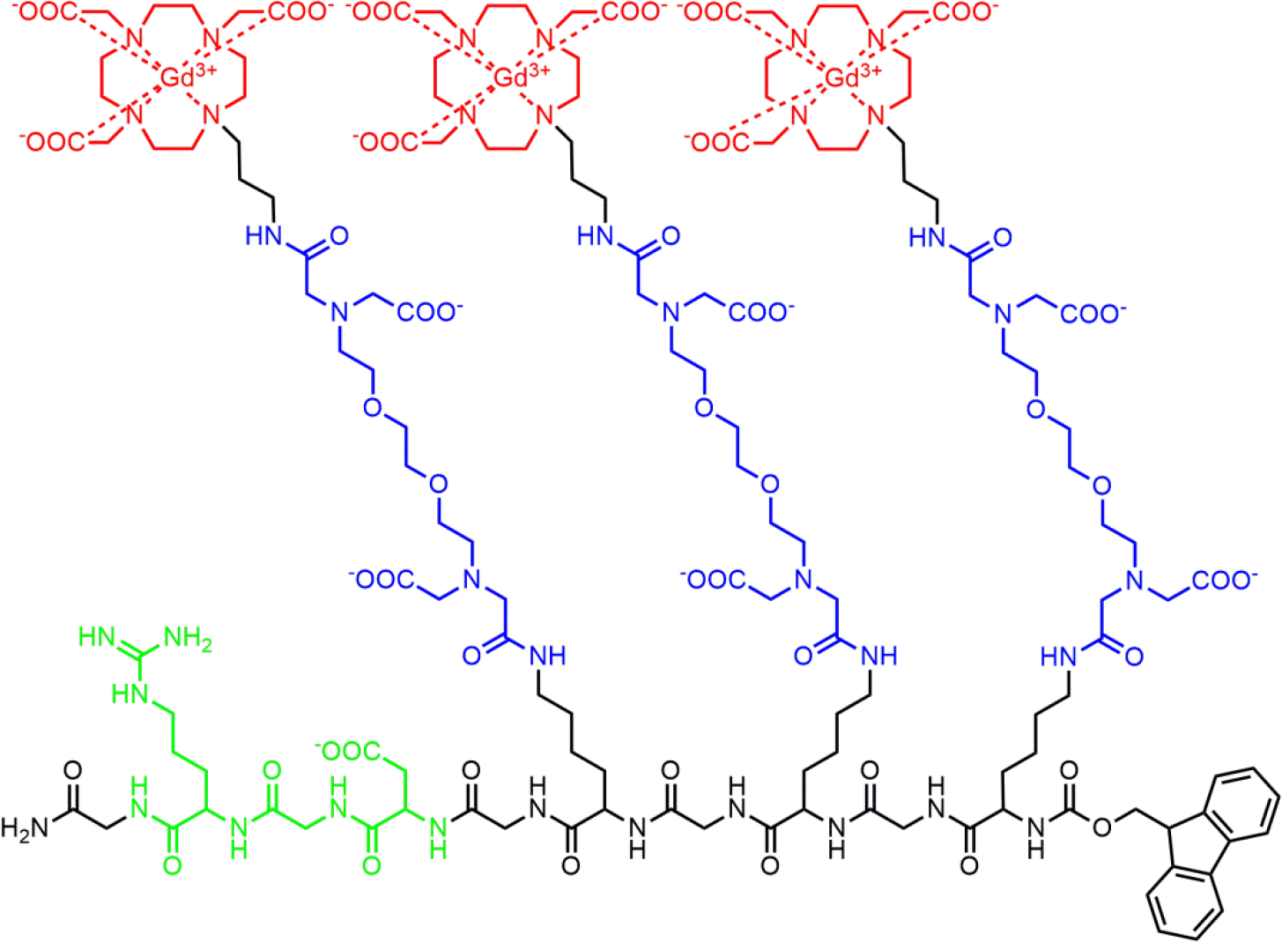
Chemical Structure of the Trimeric, RGD-Functionalized Responsive Probe **Gd**_**3**_**L**_**3**_**-RGD**, Highlighting the Gd^3+^ Chelator (red), the Ca^2+^ Chelator (blue), and the RGD-Peptide Vector (green)

The Arg-Gly-Asp (RGD) peptide sequence interaction with α_v_β_3_ integrin is a well-studied and widely exploited interaction.^26−29^ This class of transmembrane proteins plays an important role in many cell-signaling and cell–cell adhesion functions, resulting in their expression on the membranes of most cell types, including synapses.^30−32^ These features make integrins an ideal target for a molecular probe that is functionalized with a covalently bound RGD moiety. It has been successfully exploited for tumor imaging, holding great potential for the development of diverse theranostics methodologies.^33−36^ In this work, we looked at this well-established mechanism from a different perspective, developing a targeted multimeric molecular imaging probe. The RGD targeting vector and its interaction with the appropriate receptor, the integrins, would result in the slower washout of the probe from the site of injection. We used a SPPS approach to prepare and attach the desired RGD sequence as the “targeting head” of the peptide backbone to three calcium-responsive CA precursors (Scheme 1). We then investigated the relaxivity enhancement properties of the final probe in response to the coordination of Ca^2+^ by means of ^1^H NMR. Following up on the promising behavior that was observed in the relaxometric titrations, we executed a set of *in vivo* and *ex vivo* experiments. Their purpose was to verify if, by functionalizing a molecular probe with a cell-labeling peptide, it is possible to slow down the probe diffusion in the tissue.

## Results

### Solid Phase Synthesis

**Scheme 2:**
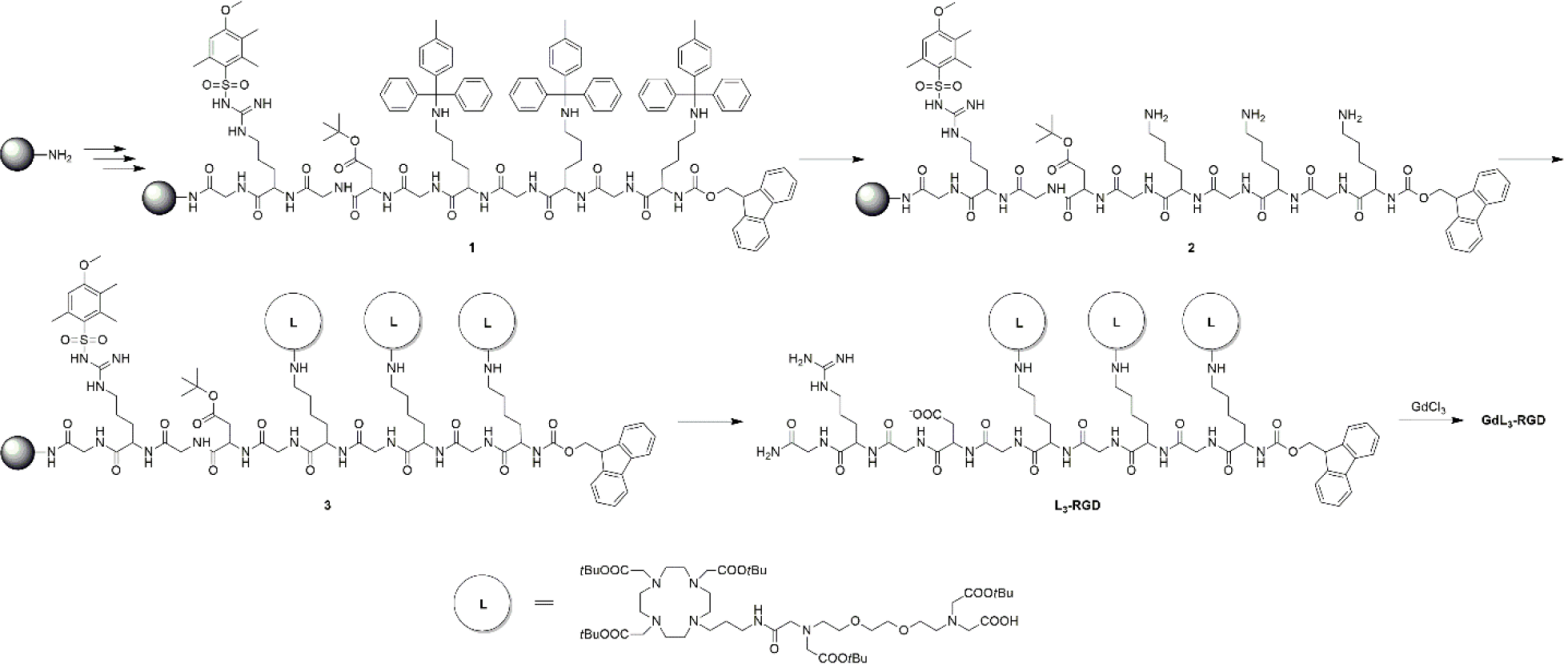
SPS Procedure for the Preparation of **Gd_3_L**_**3**_**-RGD**

The peptide was synthesized on a Rink amide resin solid support, coupling the amino acids with an amide coupling/Fmoc deprotection synthetic protocol. The final peptide sequence (GRGDGKGKGK) includes the targeting RGD sequence and three Lys residues, for coupling with the responsive agent **L** (Scheme 2), alternated with Gly units to function as spacers. The RGD targeting group was selected for its strong reported interaction with α_v_β_3_ integrin.^21,26,27,37^ In doing so, we obtained a multimeric system for cell labeling that would be characterized by a slower diffusion in the tissue of injection, owing to this strong interaction. The Ca^2+^-responsive precursor **L** was synthesized as reported previously.^38^ It was selected because its Gd^3+^ complex exhibited excellent relaxivity enhancement properties in response to the selective binding of Ca^2+^. The successful preparation of the peptide backbone was followed by a coupling step and attachment of the three **L** units to the ω-amines of the three Lys residues. The preparation of the final product with the SPPS methodology was confirmed by analytical HPLC-MS and ^1^H NMR. Following purification by semipreparative HPLC, the trimeric **L**_**3**_**-RGD** ligand was obtained. The final **Gd**_**3**_**L**_**3**_**-RGD** was obtained upon complexation with GdCl_3_, followed by treatment with Chelex for the removal of the excess of the lanthanide ion.

### Relaxometric ^1^H NMR Titrations

**Figure 1:**
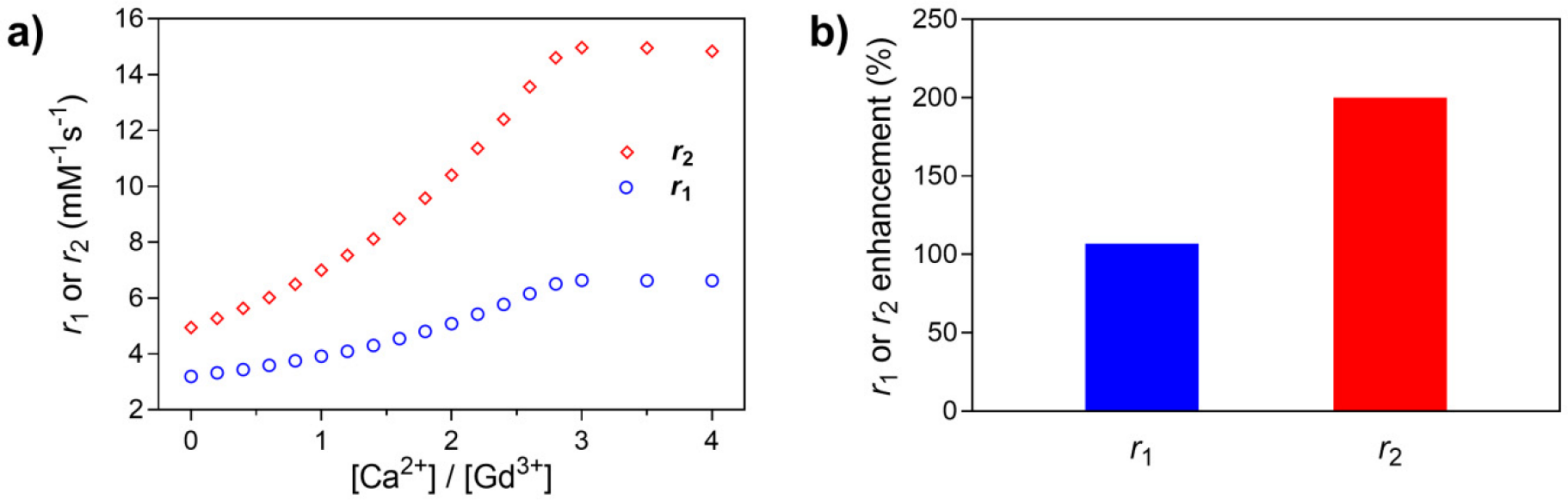
^1^H NMR relaxometric titration of **Gd**_**3**_**L**_**3**_**-RGD** with Ca^2+^: (a) *r*_1_ or *r*_2_ increase during the sequential addition of Ca^2+^; (b) overall *r*_1_ or *r*_2_ relaxivity enhancement (in %) at the end of titration.

The longitudinal and transverse relaxivity enhancement upon Ca^2+^ addition was measured at 7 T and 25 °C for a solution of **Gd**_**3**_**L**_**3**_**-RGD** with [Gd^3+^] = 1.0 mM. The initially obtained values in the absence of Ca^2+^ were *r*_1_ = 3.2 mM^–1^ s^–1^ and *r*_2_ = 4.94 mM^–1^ s^–1^ (Figure 1).

Upon saturation, the responsive probe **Gd**_**3**_**L**_**3**_**-RGD** displayed the values of 6.62 mM^–1^ s^–1^ and 14.97 mM^–1^ s^–1^ for *r*_1_ and *r*_2_, respectively, in the presence of 3.0 equiv of CaCl_2_. This corresponds to an overall enhancement of +110% and +200% in *r*_1_ and *r*_2_, respectively. The magnitude of the observed relaxivity increase is consistent with what is reported for multimeric lanthanide-based MRI CAs responsive to Ca^2+^ coordination.^39^

### *In Vivo* MRI

**Figure 2:**
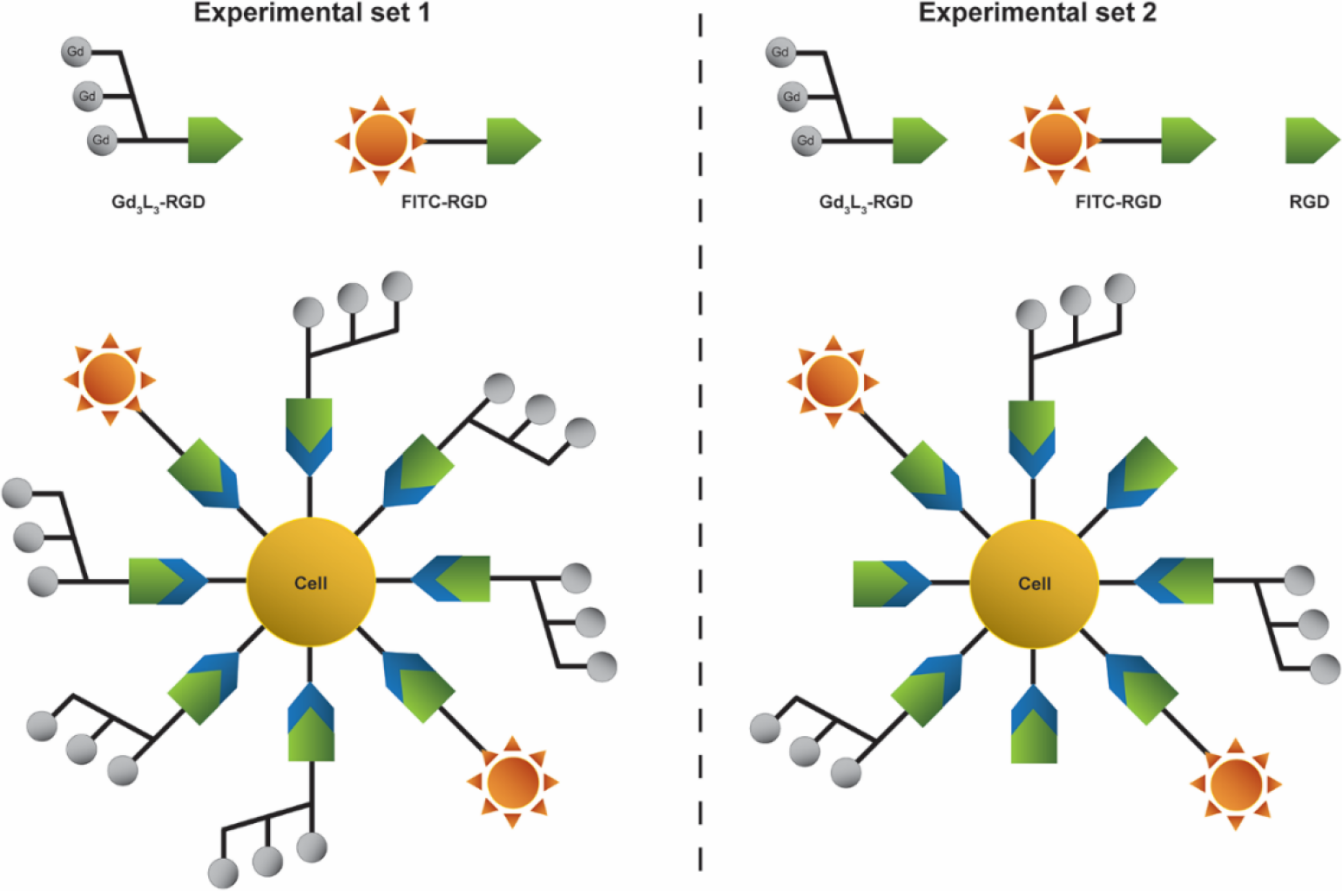
Schematic representation of the two mixtures injected *in vivo* and their interaction with the integrins expressed in the tissue.

Two sets of *in vivo* experiments on rats (*n* = 3 per set) were performed by the intracranial infusion of two different injection mixtures in the somatosensory cortex (S1). S1 is a large region with a high and homogeneous neuronal density. In addition, S1 is ideally suitable for the projected studies of combining the infusion of responsive CAs with a range of standard somatosensory stimuli that produce robust and highly reproducible calcium-dependent neuronal activations.^40^ For the first set of experiments, the injected mixture contained **Gd**_**3**_**L**_**3**_**-RGD** and a FITC-RGD, in a molar ratio of 25:1 and total [Gd^3+^] = 5.0 mM. In the second set of experiments, the injected mixture contained the same cocktail of substances used in the former experimental set, supplemented by the addition of a 5-fold excess of nonfunctionalized RGD peptide to serve as a competitor for **Gd**_**3**_**L**_**3**_**-RGD** (5-fold excess of the RGD was calculated based on the known content of Gd^3+^ in **Gd**_**3**_**L**_**3**_**-RGD**, [RGD] = ([Gd^3+^]:3) × 5 (Figure 2).

**Figure 3:**
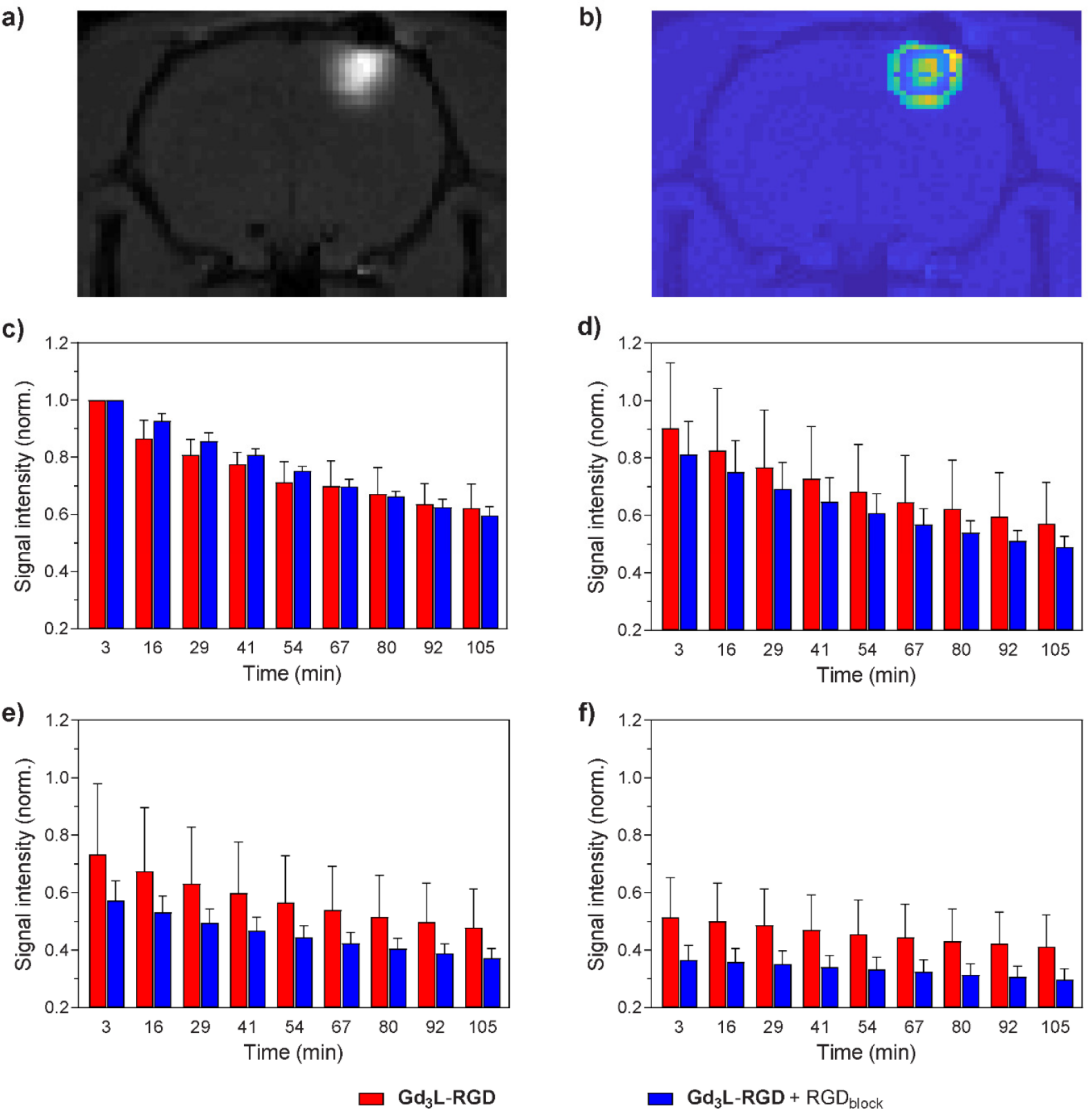
Comparison of the **Gd**_**3**_**L**_**3**_**-RGD** diffusion in the central MRI slice between the two sets of *in vivo* MRI experiments: (a) *T*_1_-weighted MRI image of the central slice, showing the COI for one of the repetitions of the experimental set 1; (b) voxel map of the ROI from the same experiment, showing the concentric voxel groups evaluated in the data analysis process; (c) averaged signals of the defined area that covers COI (500 μm diameter in the plane); (d–f) averaged signals of the defined concentric areas at 750 μm (d), 1250 μm (e), and 1750 μm (f) distance from COI. The bars indicate the normalized MRI signal intensity time profiles (*n* = 3).

Subsequently, an MRI acquisition protocol was applied to record ^1^H *T*_1_-weighted images with a 270 s interval over 3 h in a 7 T MRI scanner. The COI was easily identified due to the substantial signal enhancement caused by the presence of the contrast agent. Using the COI as a reference, three coronal slices of 1 mm thickness were defined, and the MRI signal data of their composing voxels was analyzed over time, in relation to their distance from the COI. Additionally, we evaluated the effect on the contrast enhancement performance of **Gd**_**3**_**L**_**3**_**-RGD** in the two experimental sets. This was achieved by analyzing and comparing the averaged data for each experimental set, where the voxels in the ROI were classified according to their distance from the COI (Figures 3 and S4 and S5 in Supporting Information).

The obtained MRI signals were analyzed by showing their intensity as a function of time, in order to evaluate and compare the rate of signal decrease in the two experimental setups (Figure 3). The results clearly display two distinct spatial distributions of the recorded MRI signal with respect to the COI: signal recorded in the second set of experiments (cocktail supplemented with the nonfunctionalized RGD peptide as the competitor) is weaker than in the first set (cocktail without the nonfunctionalized RGD peptide). The effect becomes more pronounced as the distance from the COI increases.

### *Ex Vivo* Fluorescence Microscopy

**Figure 4:**
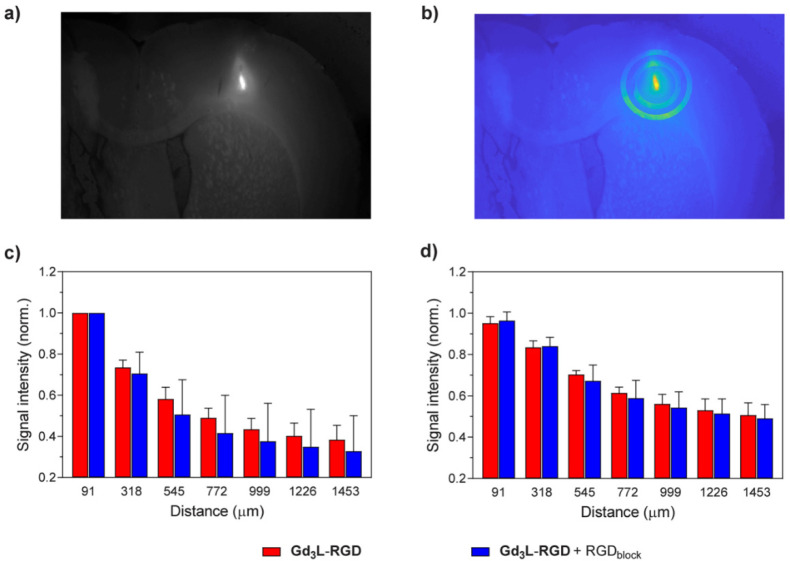
Comparison between the two sets of the fluorescence imaging experiments *ex vivo* with **FITC-RGD** on two tissue sections (slices): (a) fluorescent image of a brain slice showing the injection site; (b) a voxel map of the ROI showing the defined concentric areas evaluated in the data analysis process; (c, d) averaged and normalized signal intensity spatial profiles for (c) COI slices and (d) peripheral slices (200 μm from the COI slice).

The diffusion behavior for the two mixtures was also followed by means of epifluorescent microscopy *ex vivo*. After the *in vivo* MRI experiment was finished, the animals were euthanized, and their brains were extracted and prepared for sectioning. The obtained 200 μm thick slices were imaged individually using epifluorescence microscopy. The fluorescence signal intensity captured from the ROI was measured for the central brain slice (where the COI was), as well as its periphery (200 μm distance from COI). Consequently, we analyzed the data within each individual slice, relative to its distance from the COI itself (Figure 4). The obtained results between the two set of experiments were more similar to each other; however the former cocktail without the RGD competitor exhibited slightly higher mean signal intensities than the latter, which contained the competitor.

## Discussion

Targeted molecular probes can improve the efficacy of MRI, since its sensitivity represents one of the main hurdles for its wider application in molecular imaging. For instance, the expression of certain receptors in the tissue may often be close, if not below, the limit of detection of MRI. The use of target-specific probes, that is, systems bearing multimeric imaging probes functionalized with a single vector, can increase local concentration, which results in accumulation of the probe in the target tissue and a higher MRI signal. To this goal, we synthesized and characterized a trimeric RGD-functionalized Ca^2+^-responsive probe **Gd**_**3**_**L**_**3**_**-RGD** as a contrast agent that specifically binds to protein integrins. The synthesis of **Gd**_**3**_**L**_**3**_**-RGD** was achieved using the SPPS method, which is most commonly used for the production of peptides. This methodology represents an incredibly convenient approach to the preparation of targeted probes for molecular imaging, since it enables the synthesis of the peptidic backbone in a linear and practical fashion.^41−43^ Moreover, the obtained peptide chain can be subsequently functionalized, exploiting the side-chain functional groups of the amino acids to couple chelators for paramagnetic ions to serve as MRI-active units or ligands for targeting purposes. Thus, we exploited this convenient strategy to successfully prepare an RGD-bearing polypeptide, combined with macrocyclic units to serve as precursors for the MRI agent.

The obtained trimeric targeted CA was characterized by ^1^H NMR relaxometric titration, in order to investigate its potential as a Ca^2+^-responsive CA (Figure 1). The observed increase in *r*_1_ is due to a variation in the number of water molecules directly coordinated to the lanthanide ion, which comes as a consequence of Ca^2+^ binding.^17,44^ Additionally, the *r*_2_ response is almost 2-fold higher than the *r*_1_, as expected for a system of this category.^39^ Namely, the multimeric nature of the probe increases the structural rigidity of the monomeric units relative to each other, decreasing the local rotational freedom, hence, increasing the *r*_2_.^45^ However, the reported **Gd**_**3**_**L**_**3**_**-RGD** is to be considered a medium-molecular weight probe, if compared to the ones based on nanoparticles and smaller molecular probes on the other hand. Consequently, its increase in *r*_2_ places it in an intermediate range, between the behaviors of the aforementioned categories of responsive CAs. Notably, the measured relaxivity reaches a plateau after the addition of 3 equiv of Ca^2+^, relative to [Gd^3+^]. The extension of the saturation profile of the **Gd**_**3**_**L**_**3**_**-RGD** could be found in its peptide backbone: the binding capacity or the charge of the peptides that compose it may influence the binding of Ca^2+^ ions, changing the overall capability of the probe to coordinate Ca^2+^. This feature of **Gd**_**3**_**L**_**3**_**-RGD** is advantageous and could enable the responsivity toward the biomarker of interest to be maintained over a wider range of concentrations, particularly suitable for targeting extracellular Ca^2+^.^46^

To evaluate the **Gd**_**3**_**L**_**3**_**-RGD** as MRI targeted probe, we designed an experimental protocol *in vivo* consisting of two sets of experiments. In these, we applied two different mixtures in order to observe the effect of the RGD–integrin interaction on the *in vivo* distribution and diffusion properties of our probe. In the second set of experiments, the injected mixture contained an additional 5-fold excess of RGD as a nonfunctionalized peptide to serve as the competitor to the applied targeted **Gd**_**3**_**L**_**3**_**-RGD**, that is, blocker of the integrin receptors. We anticipated that the addition of this blocking agent can saturate a portion of integrins expressed in the tissue, thus decreasing the likelihood of an interaction between them and **Gd**_**3**_**L**_**3**_**-RGD**. Should **Gd**_**3**_**L**_**3**_**-RGD** have no targeting properties, there would be no difference observed in the diffusion profiles between these two sets of experiments. We decided for this approach as an alternative to the synthesis of an analogue to **Gd**_**3**_**L**_**3**_**-RGD** with either a scrambled peptide sequence, or the removal altogether of the peptide moiety. Such approaches would allow for a more direct assessment of the effect of the RGD–integrin interaction on the *in vivo* behavior of our targeted probe. However, the removal of the peptide tag from **Gd**_**3**_**L**_**3**_**-RGD** would represent an alteration of its molecular size and structure, while the use of a scrambled peptide sequence would have required considerable additional synthetic work for a control probe. Similarly, we opted for the inclusion of a RGD-labeled fluorescent dye rather than to couple it to the probe itself in order to maintain the structure of the probe as it would be used for *in vivo* MRI applications.

The obtained results confirmed the affirmative targeting efficacy of the RGD peptide vector toward integrins. Specifically, we measured the decay of the MRI signal over time, dividing the ROI into 4 different areas as a function of their distance from the COI (Figure 3). The MRI signal generated by **Gd**_**3**_**L**_**3**_**-RGD** is weaker in the presence of the competitor, the RGD peptide, and this effect is more pronounced as the distance from the COI increases. This could be explained by further change in the concentration ratio in more distant ROIs between the **Gd**_**3**_**L**_**3**_**-RGD** and its competitor RGD, since the small size molecule likely diffuses faster and is more abundant at a larger distance from the COI. Consequently, greater amounts of **Gd**_**3**_**L**_**3**_**-RGD** bind to the integrin receptors in the absence of the RGD blocker, which confirms the postulated behavior of **Gd**_**3**_**L**_**3**_**-RGD** as the targeting agent. This is an interesting result with a high potential for further development, particularly as the study was performed on nongenetically modified specimens. With the implementation of modern genetic engineering methodologies for studies of pathophysiological conditions that involve the overexpression of desired receptors, the potential for exploiting this probe preparation and application approach would be even greater.

The effect observed in the MRI experiments was confirmed *ex vivo* by analyzing the washout of the fluorescent dye FITC-RGD. The fluorescent images recorded for the brain slices corresponding with the COI displayed a slightly stronger fluorescence intensity in the experiments without the RGD peptide blocker, while this difference was less pronounced in the peripheral areas (Figure 4). The reason for this could be found in the experimental procedure, which required immersing the tissue for 16 h in paraformaldehyde followed by 3 days immersion in sucrose solution in order to prepare it for the fluorescence imaging experiments. It is possible that such long tissue exposure led to significant washout of the cocktail components, which resulted in the smaller differences in the obtained signal intensities between two experimental sets. However, results obtained from the fluorescence microscopy experiments are in line with those obtained by MRI, confirming that addition of the RGD peptide reduces the binding of the RGD-containing probes (**Gd**_**3**_**L**_**3**_**-RGD** or FITC-RGD), hence showing their binding capabilities to integrins.

## Conclusions

In this work, we designed and synthesized the multimeric targeted MRI responsive probe **Gd**_**3**_**L**_**3**_**-RGD** using a solid-phase supported approach for the preparation of the peptide. The multimeric complex was obtained in good yield and displayed excellent relaxivity enhancement properties upon the coordination of Ca^2+^ ions. Subsequently, *in vivo* MRI and *ex vivo* fluorescence experiments demonstrated that appending the RGD moiety to a non-nanosized probe can efficiently increase the retention of the contrast agent in the tissue. The design of targeted and responsive CAs presented in this work could enable more complex and time-demanding *in vivo* experiments to be performed, paving the way for the development of novel functional molecular imaging methodologies.

## Materials and Methods

### General Remarks

Manual solid phase synthesis was performed with the synthesis 1 apparatus from Heidolph. The Rink amide MBHA resin, 100–200 mesh, was purchased from Merck Millipore. LC-MS spectra were recorded on an Agilent 1100 series LC/MS system with a Polaris 5 C18-Ether column (250 mm × 4.6 mm). The LC-MS elution conditions are given in Table S1. Reversed-phase HPLC purification was performed on a Varian PrepStar Instrument (Australia) with PrepStar SD-1 pump heads. Analytical reversed-phase HPLC was performed with an Atlantis C18 column (4.6 mm × 150 mm, 5 μm particle size), showing the purity of products of >95%. Semipreparative reversed-phase HPLC was conducted with a Polaris 5 C18-A column (250 mm × 21.2 mm). Elution conditions are described in Table S2.

### Synthetic Procedures

The synthesis of **2** was carried out using a standard Fmoc chemistry approach with a manual peptide synthesizer. All reactions on solid phase were performed at room temperature. A Rink amide MBHA resin (0.2 g, substitution 0.78 mmol g^–1^) was selected as the solid support. Before the first amino acid was coupled, the resin was allowed to swell in DMF for 1 h, and Fmoc deprotection of the resin was carried out using a solution of 20% piperidine in DMF (3 × 15 min). Prior to each reaction, the resin was allowed to swell in DMF for 1 h. After the coupling of the first amino acid, a capping procedure using an acetic anhydride/pyridine solution (3:2, 4 mL) was performed for 30 min. The resin was then washed with DMF (5 × 3 mL). Each coupling and deprotection procedure was checked for completeness using the Kaiser test. The general procedure for coupling reactions and Fmoc deprotections are listed in the following sections.

### General Amino Acid Coupling Procedure

Fmoc-protected amino acids were dissolved in DMF (4 mL) and activated in situ with HBTU and DIPEA. After 10 min of preactivation, the mixture was added to the preswelled resin and agitated. After coupling, the resin was washed with DMF (5 × 3 mL) and CH_2_Cl_2_ (3 × 3 mL) to remove excess reagents.

### General Fmoc Deprotection Procedure

Fmoc deprotection of the resin and amino acids was carried out with treatments of a piperidine in DMF solution. After deprotection, the resin was washed with DMF (5 × 3 mL) and prepared for the next procedure.

### Peptide Synthesis Conditions

Specific reaction conditions for each coupling/deprotection procedure are provided below:

#### Resin-Gly

Coupling: Fmoc-Gly-OH (5 equiv), HBTU (4.9 equiv), DIPEA (10 equiv), 3 h. Fmoc deprotection: 20% PIP/DMF, 3 × 3 min.

#### Resin-Gly-Arg

Coupling: Fmoc-Arg(Pbf)-OH (5 equiv), HBTU (4.9 equiv), DIPEA (10 equiv), 3 h. Fmoc deprotection: 20% PIP/DMF, 4 × 3 min.

#### Resin-Gly-Arg-Gly

Coupling: Fmoc-Gly-OH (5 equiv), HBTU (4.9 equiv), DIPEA (10 equiv), 2 h. Fmoc deprotection: 40% PIP/DMF, 3 × 10 min.

#### Resin-Gly-Arg-Gly-Asp(OtBu)

Coupling: Fmoc-Asp(OtBu)-OH (4 equiv), HBTU (3.9 equiv), DIPEA (8 equiv), 4 h. Fmoc deprotection: 30% PIP/DMF, 4 × 10 min.

#### Resin-Gly-Arg-Gly-Asp(OtBu)-Gly

Coupling: Fmoc-Gly-OH (4 equiv), HBTU (3.9 equiv), DIPEA (8 equiv), 3 h. Fmoc deprotection: 40% PIP/DMF, 4 × 10 min.

#### Resin-Gly-Arg-Gly-Asp(OtBu)-Gly-Lys(Mtt)

Coupling: Fmoc-Lys(Mtt)-OH (4 equiv), HBTU (3.9 equiv), DIPEA (8 equiv), 3 h. Fmoc deprotection: 20% PIP/DMF, 3 × 10 min.

#### Resin-Gly-Arg-Gly-Asp(OtBu)-Gly-Lys(Mtt)-Gly

Coupling: Fmoc-Gly-OH (4 equiv), HBTU (3.9 equiv), DIPEA (8 equiv), 3 h. Fmoc deprotection: 20% PIP/DMF, 3 × 10 min.

#### Resin-Gly-Arg-Gly-Asp(OtBu)-Gly-Lys(Mtt)-Gly-Lys(Mtt)

Coupling: Fmoc-Lys(Mtt)-OH (4 equiv), HBTU (3.9 equiv), DIPEA (8 equiv), 3 h. Fmoc deprotection: 20% PIP/DMF, 3 × 10 min.

#### Resin-Gly-Arg-Gly-Asp(OtBu)-Gly-Lys(Mtt)-Gly-Lys(Mtt)-Gly

Coupling: Fmoc-Gly-OH (4 equiv), HBTU (3.9 equiv), DIPEA (8 equiv), 3 h. Fmoc deprotection: 20% PIP/DMF, 3 × 10 min.

#### Resin-Gly-Arg-Gly-Asp(OtBu)-Gly-Lys(Mtt)-Gly-Lys(Mtt)-Gly-Lys(Mtt)-Fmoc

Coupling: Fmoc-Lys(Mtt)-OH (4 equiv), HBTU (3.9 equiv), DIPEA (8 equiv), 3 h.

#### Resin-Gly-Arg-Gly-Asp(OtBu)-Gly-Lys(NH_2_)-Gly-Lys(NH_2_)-Gly-Lys(NH_2_)-Fmoc

The peptidyl resin was swelled in DMF for 1 h. Afterward, the resin was washed with CH_2_Cl_2_ (5 × 3 mL). The resin was then treated with a 3% TFA/CH_2_Cl_2_ (6 × 3 min) to remove the Mtt protecting groups. The peptidyl resin was then washed with DMF (5×) and CH_2_Cl_2_ (5×). The success of the reaction was then assessed by sampling a few beads and treating them with a few drops of 50% TFA/CH_2_Cl_2_ solution (colorless solution indicated the reaction was complete). The reaction was also monitored with the Kaiser test.

The resin was subject to a microcleavage procedure (same as the cleavage procedure described below) and analyzed by LC-MS.

LC-MS: (*m*/*z*) [M + H]^+^ calcd for C_53_H_82_N_17_O_14_^+^ 1180.6, found 1180.7; (*m*/*z*) [M + 2H]^2+^ calcd for C_53_H_83_N_17_O_14_^2+^ 590.8, found 590.9; (*m*/*z*) [M + 3H]^3+^ calcd for C_53_H_84_N_17_O_14_^3+^ 394.2, found 394.3; (*m*/*z*) [M – H]^−^ calcd for C_53_H_80_N_17_O_14_^−^ 1178.6, found 1178.8.

### **L_3_-RGD**

The resin (0.074 mmol) was swelled in DMF for 1 h and then agitated for 16 h with a preactivated solution of **L** (233 mg, 0.222 mmol), HATU (82 mg, 0.215 mmol), HOBt (29 mg, 0.215 mmol), and DIPEA (77 μL, 0.445 mmol) in DMF (2 mL). After the reaction, the resin was washed with DMF (5×) and CH_2_Cl_2_ (5×).

The resin was then treated with a deprotection solution composed of TFA/TIS/CH_2_Cl_2_ (95:2.5:2.5, 3 mL) for 4 h. The solution was collected, and the resin washed twice more with the deprotection solution. The filtrates were combined and a significant portion evaporated. Cold diethyl ether was then added, and the mixture was stored in the freezer overnight in order to precipitate the compound from the deprotection solution. The mixture was then centrifuged (3000*g*, 10 min), and the excess solution was removed. The precipitate was then washed with cold diethyl ether and centrifuged a further two times before being left to dry to give the crude product. The crude solid was then purified by RP-HPLC and lyophilized to give **L**_**3**_**-RGD** (24 mg). ^1^H NMR (300 MHz, D_2_O) δ (ppm): 0.82–4.56 (br, 194H), 7.13–7.96 (m, 8H). ESI-HRMS: (*m*/*z*) [M – 4H]^4–^ calcd for C_146_H_236_N_38_O_56_^4–^ 854.42023, found 854.42103; (*m*/*z*) [M – 3H]^3–^ calcd for C_146_H_237_N_38_O_56_^3–^ 1139.5627, found 1139.5632.

### **Gd_3_L_3_-RGD**

**L**_**3**_**-RGD** (22 mg, 6.43 μmol) was dissolved in water (2 mL), and the pH was adjusted to 7. A solution of GdCl_3_·6H_2_O (7.88 mg, 0.021 mmol) in water (1 mL) was added slowly while maintaining the pH at 7. The resulting solution was left to stir overnight at room temperature. Afterward, any excess Gd^3+^ was removed using rounds of treatment with Chelex (3 × 45 min), before filtering and lyophilizing to obtain **Gd**_**3**_**L**_**3**_**-RGD** as a white solid.

### Relaxometric Titrations with Ca^2+^

^1^H *T*_1_ and *T*_2_ measurements were performed at 7 T on a Bruker Avance III NMR spectrometer and 25 °C on a [Gd^3+^] = 1.0 mM solution of **Gd**_**3**_**L**_**3**_**-RGD** in HEPES buffer (50 mM) at pH 7.4. After each addition of Ca^2+^, *T*_1_ and *T*_2_ of the solution were measured and plotted against [Ca^2+^] expressed in equivalents ([Ca^2+^]/[Gd^3+^]) to obtain the titration profiles.

From the titration profile, the millimolar relaxivities, *r*_1_ and *r*_2_, were calculated according to the eq 1, where *T*_*i*,obs_ and *T*_*i*,d_ are recorded (observed) and background (diamagnetic) *T*_1_ or *T*_2_ relaxation times, respectively, while [Gd^3+^] is concentration of Gd^3+^ at each point of the titration experiment.
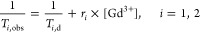
1

### MRI *In Vivo* Experiments

MRI measurements were performed on a Bruker BioSpec 70/30 USR magnet (software version Paravision 5.1) using a transmit–receive ^1^H Bruker volume coil (RF RES 300 1H 075/40 QSN TR).

*T*_1_-weighted MR images were acquired using the FLASH pulse sequence, performed continuously for 172.2 ± 0.8 min using the following parameters: repetition time = 77.4 ms, echo time = 2.81 ms, flip angle = 90°, number of averages = 24, acquisition time = 3 min 11 s, field of view = 30 mm × 25.8 mm, matrix size = 120 × 103, spatial resolution 250 μm × 250 μm, slice thickness 1 mm, number of slices 3.

Animal experiments were conducted on male Sprague–Dawley rats (320–370 g, Charles River Laboratories). Animals were housed and maintained in controlled environmental conditions with 12:12 h light–dark cycle for at least 7 days before the experiment, with food and water provided ad libitum. All experiments with animals were done in accordance with EU Directive 2010/63/EU on the protection of animals used for scientific purposes and were approved by the local authorities (Regierungspräsidium Tübingen).

After the animal induction with 2.5% isoflurane in O_2_ (Forene, Abbott, Wiesbaden, Germany), the animal was fixed in a stereotaxic device (Stoelting Co., IL, US), and the inhalation was switched to a mixture of oxygen and nitrous oxide (1:2) to induce analgesia. After ensuring deep anesthesia by the absence of pedal withdrawal, the surgical procedure was initiated. The craniotomy was performed using a manual drill (ML = 3.3, AP = 0.2, DV = 2), and the dura was removed. Thereafter, the injection mixture (1 injection set, **Gd**_**3**_**L**_**3**_**-RGD** and FITC-RGD 25:1, or 2 injection set, **Gd**_**3**_**L**_**3**_**-RGD**, FITC-RGD and RGD 25:1:125 in PBS, 5 mM [Gd^3+^]) was delivered intracranially at a rate of 200 nL/min for a total volume of 4 μL using a 5 μL Hamilton syringe attached to a precision pump (70-4507, Harvard Apparatus). The body temperature of the animal was maintained at 37.0 ± 0.5 °C while monitored with a rectal probe with a feedback controlled heat pad (50-7221-F, Harvard Apparatus, MA, US). After surgery, the animal was transferred to the MRI scanner. There, the inhalation mixture was changed to a mixture of air (800 mL/min) and oxygen (50 mL/min), and body temperature was monitored with a rectal probe and kept around 37 °C using a water bath. Oxygen saturation and heart rate were monitored throughout the surgery and the experiment with a pulse oximeter (MouseOx, Starr Life Sciences).

### *Ex Vivo* Fluorescence Microscopy

The animal was kept in the same animal bed holder that was used for the MRI after euthanasia. Using a circular drill head, the upper part of the skull was removed. Exposing the animal’s brain, a custom-built dissection tool was used to dissect out the region where the contrast agent was injected. Thereafter, the selected brain region was immersed in paraformaldehyde overnight (no light, 4 °C). The next day, the sample was transferred into a 30% sucrose–water solution, where it was kept for 3 days (no light, 4 °C). Lastly, using a sliding microtome (Microm HM-450, ThermoFischer Scientific), the sample was cut into 200 μm thick axial slices in anterior–posterior direction, starting with the contralateral side, that were consequently imaged individually using epifluorescence microscopy (AxioZoom V16, Carl Zeiss GmbH, Goettingen, Germany; EGFP ET Filterset, AHF analysentechnik AG, Tuebingen, Germany) with a high-resolution HRm Rev.3 (4164 × 3120 pixel resolution, 4.54 μm × 4.54 μm pixel size; Carl Zeiss GmbH, Goettingen, Germany).

### Data Analysis

#### MRI

*T*_1_-weighted images were scaled and converted to Nifti format. Sinc interpolation from the flirt program in FSL (v 5.0) package was used for motion correction.^47^ Motion was estimated from interleaved *T*_1_-weighted images and motion correction was conducted using FSL. Thereafter, the images were reconstructed and analyzed using an in-house software written in Matlab (R2018a).

For each data set (set of 38 *T*_1_-weighted images), coordinates for center of injection were manually selected. From there, area of an inner circle (1 voxel radius) followed by areas of 3 concentric circles, having thickness of 2 voxels, were defined. For all the areas individually, average signal intensity was calculated, including only voxels above the threshold value. This value was calculated using the mean value of a manually selected ROI from the same area on the contralateral side of the animal’s brain. Lastly, the obtained data was normalized for each data set to the average signal intensity value of the inner circle for the initial *T*_1_-weighted image.

#### Fluorescence Microscopy

Analysis for the fluorescence images were performed in the same way as described above for MR images, with the defined radius for inner circle of 20 voxels (90.8 μm), followed by the areas of 7 concentric circles with thickness of 50 voxels (227 μm).

## Supporting Information Available

LC-MS analysis, HRMS and ^1^H NMR of **L**_**3**_-**RGD**, comparison of the **Gd**_**3**_**L**_**3**_-**RGD** diffusion *in vivo* in two additional MRI slices (PDF)
Molecular formula strings (CSV)

## Abbreviations Used

CA: contrast agent
COI: center of injection
DIPEA: *N*,*N*-diisopropylethylamine
FITC-RGD: RGD-labeled fluorescein isothiocyanate dye
FLASH: fast low angle single shot
HATU: 1-[bis(dimethylamino)methylene]-1*H*-1,2,3-triazolo[4,5-*b*]pyridinium 3-oxide hexafluorophosphate
HBTU: (2-(1*H*-benzotriazol-1-yl)-1,1,3,3-tetramethyluronium hexafluorophosphate
HEPES: 4-(2-hydroxyethyl)piperazine-1-ethanesulfonic acid
HOBt: hydroxybenzotriazole
MBHA: 4-methylbenzhydrylamine
PIP: piperidine
ROI: region of interest
SPPS: solid phase peptide synthesis
TIS: triisopropylsilane
