# Supplementary material for: RGD-Peptide Functionalization Affects the In Vivo Diffusion of a Responsive Trimeric MRI Contrast Agent through Interactions with Integrins
Source: J Med Chem. 2021 May 7;64(11):7565–74. doi: 10.1021/acs.jmedchem.1c00264 (PMC8279402; doi:10.1021/acs.jmedchem.1c00264)
Supplement: Supplementary file 1 — jm1c00264_si_001.pdf [file jm1c00264_si_001.pdf]

Supporting Information for

RGD-peptide functionalization affects the *in vivo* diffusion of a responsive trimeric MRI contrast agent through interactions with integrins

Giuseppe Gambino[1], Tanja Gambino[1], Liam Connah[1], Francesca La Cava[1], Henry Evrard[1,2,3] and Goran Angelovski[1,4,*]

1. Max Planck Institute for Biological Cybernetics, Department for Physiology of Cognitive Processes, Max Planck Ring 11, 72076 Tübingen, Germany.
2. Nathan S. Kline Institute for Psychiatric Research, 140 Old Orangeburg Road, Orangeburg, NY 10962, USA.
3. Werner Reichardt Centre for Integrative Neuroscience, University of Tübingen, Otfried-Müller-Strasse 25, 72076 Tübingen, Germany.
4. Laboratory of Molecular and Cellular Neuroimaging, International Center for Primate Brain Research (ICPBR), Center for Excellence in Brain Science and Intelligence Technology (CEBSIT), Chinese Academy of Sciences (CAS), Shanghai 201602, PR China.

* Email: goran.angelovski@tuebingen.mpg.de

Contents

LC-MS analysis S2

HRMS spectrum of **L_3-RGD** S4

^{1}H NMR spectrum of **L_3-RGD** S4

Gd_3L_3-RGD diffusion profiles in the anterior and posterior MRI slices S5

LC-MS analysis

Table S1. Elution conditions for LC-MS analysis. A flow rate of 1 mL/min was used. Solvent A = 0.1 % CH_3COOH/H_2O. Solvent B = 0.1 % CH_3COOH/CH_3CN.

Time (min)	A %	B %
0	95	5
10	80	20
20	20	80
25	0	100
30	0	100
31	95	5
33	95	5

Table S2. Elution conditions for analytical and semi-preparative HPLC. Flow rates of 1 and 10 mL/min were used respectively. Solvent A = H_2O. Solvent B = CH_3CN.

Time (min)	A %	B %
0	95	5
5	95	5
20	0	100
39	0	100
40	95	5

a)

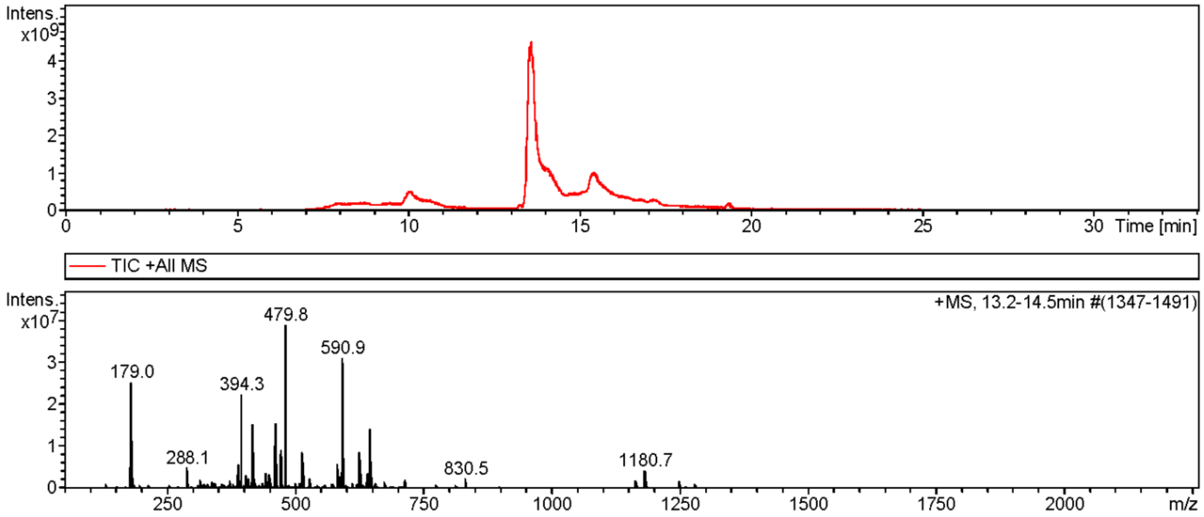

b)

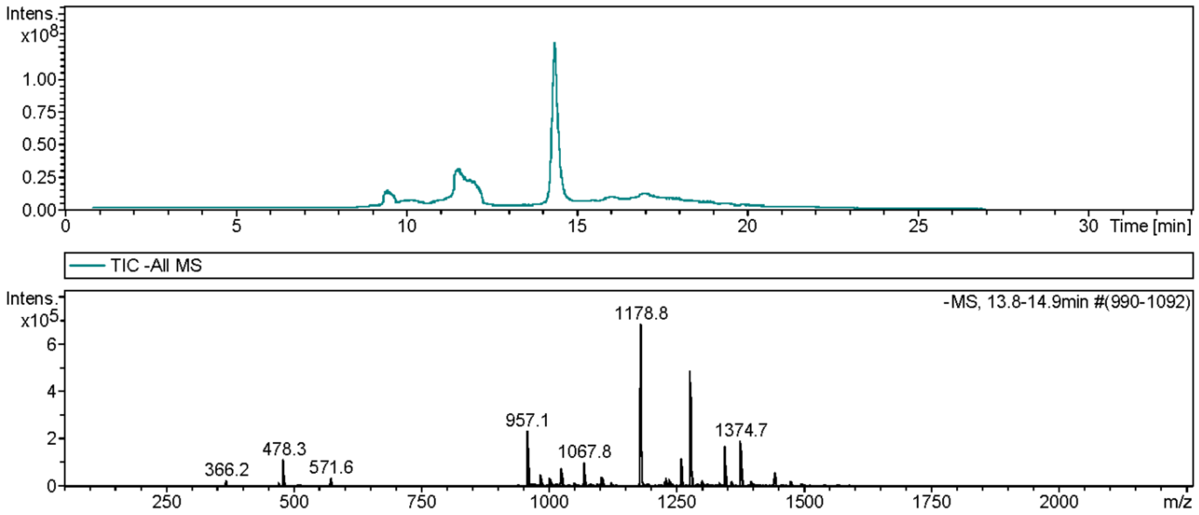

Figure S1. LC-MS traces of the product obtained from the microcleavage procedure of **Resin-Gly-Arg-Gly-Asp(O*t*Bu)-Gly-Lys(NH_2)-Gly-Lys(NH_2)-Gly-Lys(NH_2)-Fmoc** in: a) positive and b) negative ionization mode.

HRMS spectrum of L3-RGD

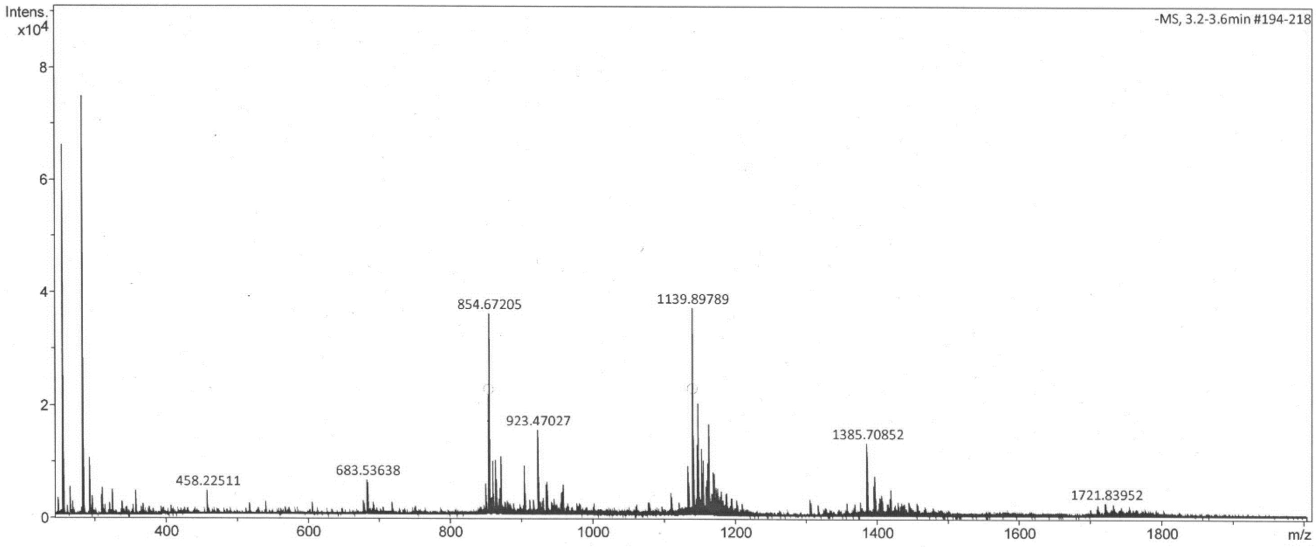

Figure S2. HRMS spectrum of **L3-RGD**.

^{1}H NMR spectrum of L3-RGD

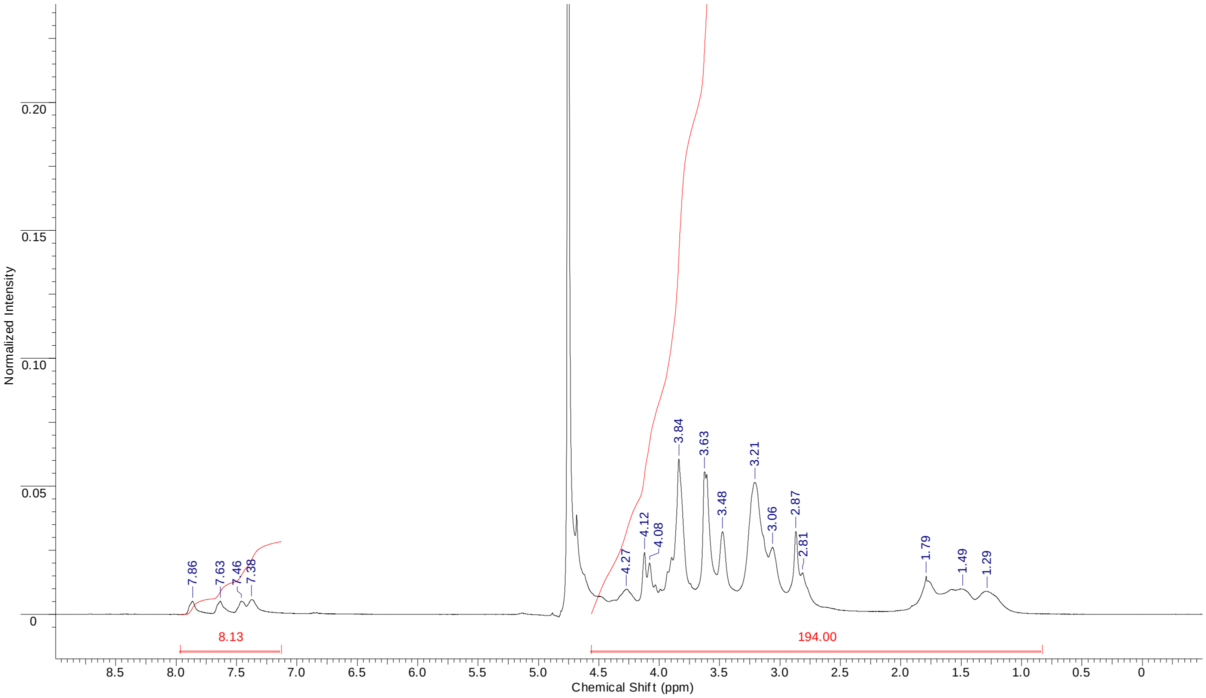

Figure S3. ^{1}H NMR spectrum of **L3-RGD** (300 MHz, D_2O).

Gd_3L_3-RGD diffusion profiles in the anterior and posterior MRI slices

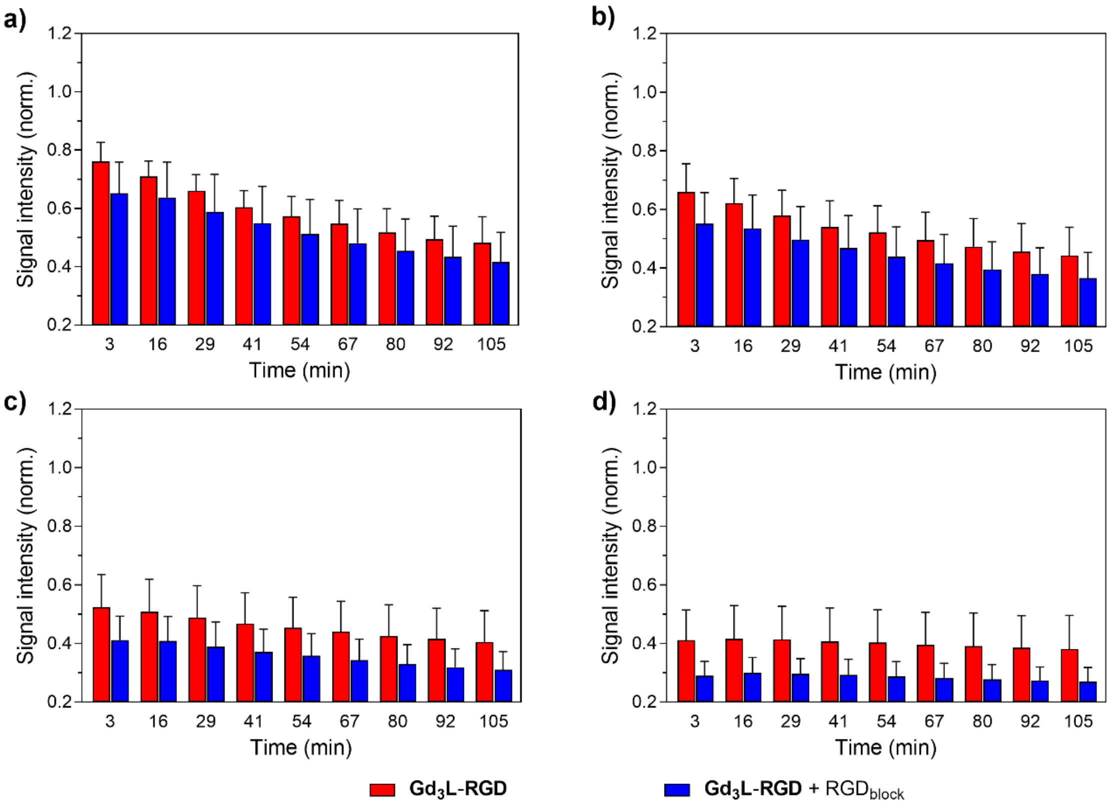

Figure S4. Comparison of the **Gd_3L_3-RGD** diffusion in the anterior MRI slice between the two sets of *in vivo* MRI experiments. a) averaged signals of the area of injection site (500 µm diameter in the plane); b-d) averaged signals of concentric areas at 750 µm (b), 1250 µm (c) and 1750 µm (d) distance from COI. The bars indicate the normalized MRI signal intensity time profiles (n = 3).

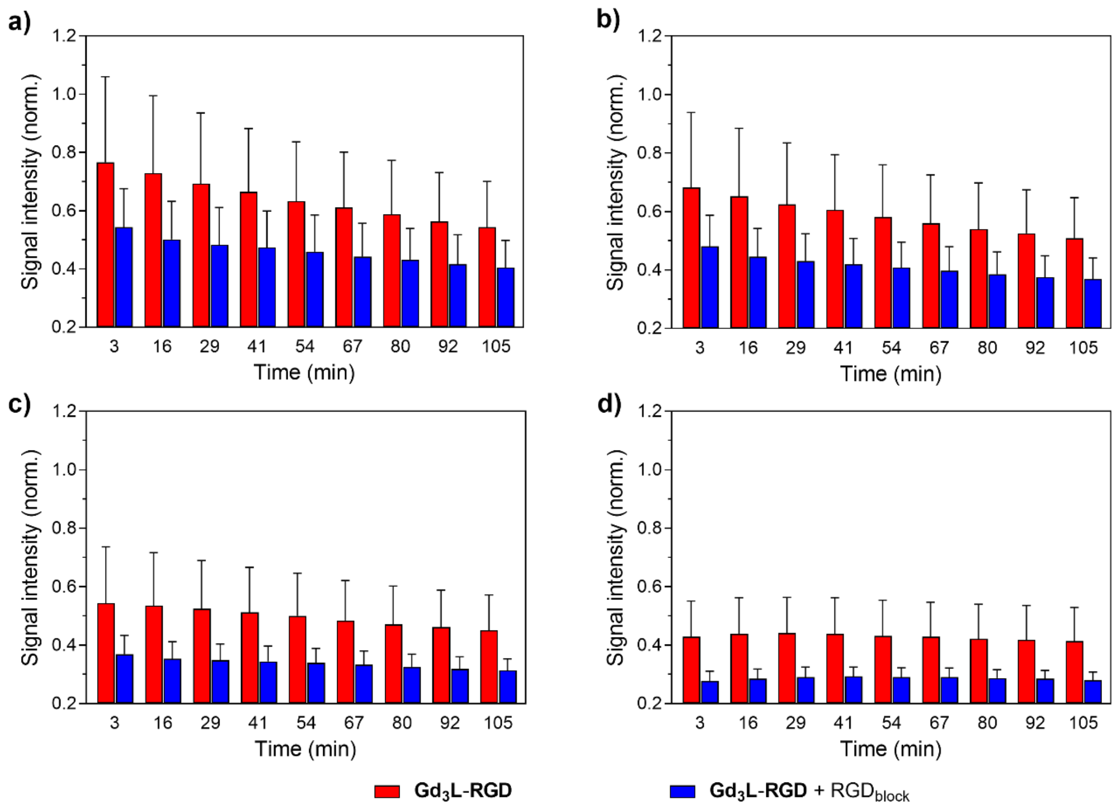

Figure S5. Comparison of the **Gd3L3-RGD** diffusion in the posterior MRI slice between the two sets of *in vivo* MRI experiments. a) averaged signals of the area of injection site (500 µm diameter in the plane); b-d) averaged signals of concentric areas at 750 µm (b), 1250 µm (c) and 1750 µm (d) distance from COI. The bars indicate the normalized MRI signal intensity time profiles (n = 3).
